# Association of Diverse Genotypes and Phenotypes of Immune Cells and Immunoglobulins With the Course of HIV-1 Infection

**DOI:** 10.3389/fimmu.2018.02735

**Published:** 2018-11-26

**Authors:** Liuzhe Li, Yan Liu, Miroslaw K. Gorny

**Affiliations:** ^1^Department of Pathology, New York University School of Medicine, New York, NY, United States; ^2^Institute of Pathogenic Biology, Medical College, University of South China, Hengyang, China

**Keywords:** HIV-1, HIV-1 disease progression, immune cells, surface proteins, immunoglobulin, genotype, phenotype

## Abstract

Disease progression among HIV-1–infected individuals varies widely, but the mechanisms underlying this variability remains unknown. Distinct disease outcomes are the consequences of many factors working in concert, including innate and adaptive immune responses, cell-mediated and humoral immunity, and both genetic and phenotypic factors. Current data suggest that these multifaceted aspects in infected individuals should be considered as a whole, rather than as separate unique elements, and that analyses must be performed in greater detail in order to meet the requirements of personalized medicine and guide optimal vaccine design. However, the wide adoption of antiretroviral therapy (ART) influences the implementation of systematic analyses of the HIV-1–infected population. Consequently, fewer data will be available for acquisition in the future, preventing the comprehensive investigations required to elucidate the underpinnings of variability in disease outcome. This review seeks to recapitulate the distinct genotypic and phenotypic features of the immune system, focusing in particular on comparing the surface proteins of immune cells among individuals with different HIV infection outcomes.

## Introduction

People infected with HIV-1 exhibit remarkable variation in disease progression. The outcome of HIV-1 infection is categorized into three groups: (a) long-term non-progressors (LTNPs), who can be subdivided into elite controllers (those with CD4^+^ T cell counts >500/mm^3^, undetectable viral load [VL], and spontaneous control of HIV-1 without antiretroviral therapy [ART] for more than 7 years and virus controllers (those who establish and retain effective control of HIV-1 for <7 years); (b) typical progressors (TPs); and (c) rapid progressors (RPs). About 5–15% of all HIV-1–infected individuals are LTNPs, and fewer than 1% of all HIV-1–infected individuals are elite (1, 2). In TPs and RPs who do not receive treatment, CD4^+^ T-cell counts fall below 200/mm^3^, and VLs rise above 10,000 copies/mL. In the absence of intervention, TPs progress to AIDS within 3–7 years, and RPs in <3 years (3).

The outcome of HIV-1 infection depends on both viral and host factors; the latter include features of cellular (CD4 and CD8 cells), humoral (B cells), and innate immunity (NK cells). Based on multiple analyses, it is clear that both genotypes (e.g., HLA) and phenotypes (e.g., CD73) contribute to the eventual outcome of HIV-1 infection in both LTNPs and progressors. Studies of HIV-1–infected individual pairs in which the same replication-competent viruses from elite controllers resulted in progressive disease in other patients have also provided solid evidence for the roles of the host genetic and phenotypic factors (4, 5). Modern clinical medicine is moving in the direction of personalized medicine based on the individual patient's response to treatment. The ontogeny based strategies for HIV vaccine design was recently emphasized by researchers (6).

In this review, we focus on comparing the genotypes and phenotypes of immune cell–surface proteins and immunoglobulins (Igs), which constitute the first line of interaction between host and virus, among individuals with different HIV infection outcomes.

## Results

### HLA genes associated with favorable and unfavorable outcomes of HIV infection

Human leukocyte antigen (HLA) alleles play vital roles in the cellular immune response to HIV infection. Several HLA alleles protect against or promote susceptibility to HIV-1 infection (7, 8). The protective or sensitizing effects of HLA genes are mediated by cytotoxic T lymphocytes (CTLs) capable of recognizing a broad spectrum of epitopes: the protective alleles enable these cells to kill HIV-infected target cells (7), whereas sensitivity can result from inhibitory effects on NK cells (9).

HLA-B^*^57, the most widely recognized protective allele (10–14), is strongly associated with slow HIV-1 disease progression in multiple ethnicities, including Caucasians (15) and African Americans (16). In a cohort study, 29 of 66 (44%) elite and 20 of 60 (33%) virus controllers carried HLA-B^*^57, whereas only 10% of chronic progressors carried this allele (17). The protective HLA-B^*^57 allele is associated with the induction of a restricted CTL response (18). In mechanistic terms, the protective effects of the HLA-B^*^57 allele relate to the breadth, magnitude, immunodominance, and affinity of the T-cell response. The peptide-binding groove of the MHC molecule facilitats the negative thymic selection of T cell clones due to fewer self-peptides able to bind to HLA-B^*^57 molecules, it leads to higher precursor frequency for viral epitopes, which would result in more cross-recognition of viral epitope variants and greater response magnitude to viral epitopes (19). HLA-B^*^57 molecules have an intrinsic preference for presenting epitopes from p24, one of the most conserved regions of the HIV genome, further contributing to improved cross-recognition (20). HLA-B^*^57–restricted CTL responses exhibit exceptionally high affinity and dominate the HLA-A^*^02–restricted CTL response in individuals co-expressing both alleles (21). Furthermore, HLA-B^*^57 molecules engage in cross-talk with innate immune cells by acting as ligands for the KIR3DL1 family of killer immunoglobulin-like receptors (KIR) on natural killer (NK) cells; these NK cells exhibit greatest multi-functionality among NK cells from subjects with different HLA-B alleles (22).

Another HLA genotype associated with control of HIV infection is HLA-B^*^27 (23, 24), which is significantly overrepresented among individuals that progress slowly to AIDS. As with HLA-B^*^57, HLA-B^*^27–restricted CTLs play a crucial role in controlling HIV-1 by presenting HIV-1 conserved epitopes of Gag p24 (25). However, in contrast to HLA-B^*^57, HLA-B^*^27-restricted CTL responses do not have particularly high affinity and do not dominate the response in individuals co-expressing HLA-A^*^02. On the contrary, HLA-B^*^27 enhances the responsiveness of HIV-specific T cells restricted through HLA-A^*^02, implying a distinct mechanism (21).

For ethnicities in which HLA-B^*^57 and HLA-B^*^27 are uncommon, other HLA-B alleles play protective roles. For example, HLA-B^*^6701 and HLA-B^*^5201-C^*^1202 haplotypes are protective alleles found in Japanese individuals with HIV infection, CTLs restricted by these alleles control HIV-1 infection progress (26, 27). Similarly, HLA-B^*^5801 and HLA-B^*^3505 mediate protective effects in Africans (28) and Thai patients infected with subtype CRF01_AE (29).

Both HLA-C alleles (30–32) and the level of HLA-C expression are associated with HIV control (12, 33, 34). HLA-C^*^1403 and HLA-C^*^1202 confer protective effects in chronically infected Japanese (32) and subtype AE–infected Vietnamese patients (31). A single-nucleotide polymorphism (SNP) in the HLA-C gene affects post-infection outcome by influencing surface expression of HLA-C protein (35). Induction of HLA-C expression by SNP rs9264942 −35 C is associated with lower HIV-1 VLs in ART-naive individuals, whereas −35 T allele, which decreases surface expression of HLA-C, is associated with relatively rapid disease progression (13, 34, 36). The protective effects of HLA-C can be mediated either by ligand-receptor interactions with KIRs (32), by induction of superior cytotoxic T cell activity via antigen presentation to cytotoxic T cells (37, 38), or likely by linkage disequilibrium with protective HLA-B alleles (39, 40).

Other HLA class I alleles associated with resistance to HIV or slower progression to AIDS include HLA-A^*^02 (41), HLA-A^*^11(42), HLA-A^*^7401 (43), HLA-B^*^18 (44), HLA-B^*^40 (44), HLA-B^*^81 (45), HLA-B^*^1402 (46), HLA-B^*^5101(47), and HLA-C8 (24). HLA-A^*^02 decreases the risk of HIV transmission to infants during childbirth by 9-fold, possibly by stimulating peripheral blood mononuclear cells that inhibit HIV replication (48). Notably, in the RV144 HIV-1 vaccine trial, carriers of HLA A^*^02 exhibited better vaccine efficacy than non-carriers (74% vs. 15%, *P* = 0.02) against viruses harboring K169; this site is essential to antibody binding, implying that immune pressure contributed to this effect (49). HLA-B^*^18 is also associated with protection against mother-to-child HIV-1 transmission: infants with HLA B^*^18 are 74% less likely to be infected at the age of 1 month, and no uninfected breastfeeding infants expressing HLA B^*^18 at 1 month subsequently acquire HIV-1 via the breast milk (50).

Unexpectedly, HLA-A^*^02 haplotypes such as HLA-A^*^02-Cw^*^16 and HLA-A^*^02-B^*^45- Cw^*^16 appear to contribute to higher VLs in HIV-infected Zambians (51). HIV has evolved to evade immune recognition by several mechanisms. For example, the viral accessory protein Nef binds to the cytoplasmic tail of class I HLA-A and B molecules, causing them to migrate to the lysosomes for degradation; this prevents surface expression of HLA molecules and thereby impairs CTL recognition of virus-infected cells (52, 53). In addition, HLA-B^*^35Px (54), HLA-B^*^08 (8), and HLA-A^*^24 alleles (55) are associated with relatively rapid progression to AIDS. Infants carrying HLA-A^*^29 are at 2-fold greater risk of acquiring HIV acquisition: in one study, 13 (25%) of 52 infants expressing HLA A^*^29 became infected by month 1, in comparison with 52 of 381 (13.7%) without this allele (50). Moreover, class I HLA-B^*^7 is correlated with accelerated disease progression in B-clade infection, but not in C-clade infection (56).

Allele-specific interactions between HLA class I molecules and their receptors on dendritic cells can significantly influence HIV-1 disease outcomes (57). Carriers of HLA-B^*^35 exhibit marked differences in resistance or vulnerability to HIV infection. Carriers of certain subtypes of HLA-B^*^35 progress more rapidly to HIV disease due to an interaction between HLA class I and inhibitory leukocyte immunoglobulin-like receptors (LILRs) expressed on dendritic cells, which leads to impaired dendritic cell function (57). HLA-B^*^35 alleles can be classified into B^*^35-Px and B^*^35-Py subtypes. HLA-B^*^35-Px molecules bind peptides with a proline (P) at anchor residue 2, and accommodate a range of residues at position 9, whereas HLA-B^*^35-Py molecules bind peptides with a proline at residue 2 but only when tyrosine (Y) is present at position 9 (58). In contrast to non-HLA-B^*^35-Px subtypes, HLA-B^*^35-Px subtypes (B^*^3502, B^*^3503, B^*^3504, and B^*^5301) are associated with faster HIV-1 disease progression (*P* < 0.0001) and have significantly higher mean HIV RNA set points (*p* = 0.04) in infected individuals in the United States and Europe (54). The putative HLA-B^*^35-Py allele B^*^3505 is protective in Thais infected with subtype CRF01_AE, a population in which the frequency of HLA-B^*^57 is low (29). However, the protective effect is not consistent across ethnicities: in a Peruvian MSM cohort, it was associated with increased VL (59).

Immune responses to HLA-B^*^35-Px– or HLA-B^*^35-Py–restricted HIV-1–specific CTL epitopes exhibit different patterns. Measurements of the immune response to variant peptides reveal that HLA-B^*^35-Py carriers do not recognize variant epitopes alone. Conversely, all HLA-B^*^35-Px carriers, who are expected to have limited recognition of epitope variants, are able to respond to all variants (60). Thus, the protective effect of HLA-B^*^35-Py may be compensated by other mechanisms.

During chronic HIV-1 infection, immunoglobulin-like transcript 4 (ILT4), a prominent inhibitory myelomonocytic MHC class I receptor expressed primarily on monocytes and dendritic cells, is significantly up-regulated (57). *Ex vivo* assessments revealed that HLA-B^*^3503 binds to ILT4 more strongly than HLA-B^*^3501, independent of the epitopes presented, leading to greater functional impairment of dendritic cells. However, HLA-B^*^3501-mediated protection from HIV-1 infection is not uniquely due to lower-affinity binding to ILT4, and may also be a result of the altered breadth of the CD8^+^ T cell response. Subjects with HLA-B^*^3501 more effectively controlled C clade infection than B clade infection, because of polymorphism in gag epitopes which were weakly recognized by CD8 cells (61). Nevertheless, in another large HIV-1–infected cohort in Mexico (62), HLA-B^*^3501 had a significant negative influence on plasma VL.

The deleterious effect of elevated expression of HLA-A on virus and CD4^+^ T-cell has been observed in 9763 HIV-infected individuals from 21 cohorts. The negative impact is mediated by elevated expression of HLA-E, which serves as a ligand for the inhibitory NK cell receptor NKG2A; the resultant increase in NKG2A-mediated NK (and/or T-cell) inhibition impairs elimination of HIV-infected target cells (9). Homozygous carriers of HLA-A,-B, and -C confer a significant risk of accelerated infection due to the smaller range of class I molecules available for antigen presentation to CTLs in comparison to heterozygous carriers (63). In general, HLA-B may be more protective against HIV-1 than HLA-A because it confers greater resistance to Nef-mediated down-regulation of HLA-I surface expression (64).

For HLA-class II, several DQB1 alleles and DQ haplotypes are associated with resistance or susceptibility to HIV-1 infection. In an association study of a cohort of 978 sex workers in Kenya, resistance-relevant alleles included DQB1^*^0603, DQB1^*^0609, DQB1^*^050301, and the DQA1^*^010201-DQB1^*^0603 haplotype; these associations were inferred from HIV-1–resistant women who remained HIV seronegative for at least 3 years while continuing active sex work (65). On the whole, DQB1^*^0602 and the DQA1^*^010201-DQB1^*^0602 haplotype were significantly overrepresented in the HIV-1–infected population. Moreover, haplotypes DQA1^*^0504-DQB1^*^0201, DQA1^*^010201-DQB1^*^0201, DQA1^*^0402-DQB1^*^0402, and DQA1^*^0402-DQB1^*^030101 were only found in HIV-1–positive women who seroconverted rapidly (65). Another analysis comparing HLA-DQ marker frequencies between the regional control population (98 African Americans, 143 Caucasians) and the disease population (30 African Americans and 22 Caucasians) linked HLA-DQB1^*^0603 (*p* = 0.04) with protection in Caucasians. By contrast, a significantly increased risk of HIV infection is associated with HLA-DQB1^*^0602 (*p* = 0.04) in Caucasians, and with HLA-DQB1^*^0605 (*p* = 0.05) in African Americans (66). In a Spanish cohort of HIV-exposed seronegative (HESN) individuals, DQB1^*^0302 was associated with immune protection against HIV infection (67). In addition, Class II HLA-DR2 is associated with susceptibility to HIV infection (42) and pulmonary tuberculosis (PTB) in Indians (68).

The association between HLA and HIV infection outcome has been extended to analyses of HIV-1 superinfection. Elevated HIV-1 superinfection risk has been observed in carriers of HLA-B^*^35 (*p* = 0.017; *P* = 0.020), HLA-C^*^04 (*p* = 0.010; *P* = 0.033), and HLA-DRB1^*^08 (*p* = 0.011; *P* = 0.027) using univariate and multivariate analyses (69).

In summary, various HLA alleles have been associated with either protective or deleterious impacts on HIV infection outcome (Table 1). The mechanisms underlying these effects include restricted CTL responses via the intrinsic breadth and affinity of HLA molecules for viral epitopes, cross-talk with NK cells as ligands to activate or inhibit innate immune cells, and the influence of cell-surface expression of HLA molecules on antigen presentation. The beneficial and deleterious effect of each HLA allele has not yet been comprehensively elucidated, in part due to the properties of the HIV-specific CTL reaction and the complexity of HLA allele profiles in individuals. In addition, in contrast to the solely protective effects of HLA-B^*^57 and B^*^27 reported in multiple studies, other alleles, such as HLA-A^*^02 and HLA-B^*^35 Px/Py subtypes, have opposing effects on disease progression, as demonstrated in studies in which HLA alleles have to be evaluated in specific genetic background in order to detect synergies or counteracting effects. HLA alleles can either act independently or function together. Accordingly, the influence of HLA alleles on disease outcome must be determined via various types of genetic dissection.

**Table 1:** HLA alleles and chemokine receptor genotypes relevant to the outcome of HIV-1 infection.

	**Genes**	**Phenotypes**	**References**
HLA	B*57^a^	Slow	(10–22, 54)
	B*27^a^	Slow	(21, 23–25, 54)
	B*6701, B*5201-C*1202	Slow	(26, 27)
	B*5801	Slow	(28)
	C1 C*1403 C*1202^b^	Slow Suppression of viral replication Lower pVL and higher CD4 counts	(30) (32) (31, 32)
	C: SNP(-35 C)^a^	Slow	(12, 13, 33–35, 38)
	C: SNP(-35 T)	Rapid	(13, 34, 36, 38)
	A*02^b^	Resistance, Lower risk in Mother-Child transmission	(41, 48)
	A*11 A*7401	Resistance Slow	(42) (43)
	B*18	Slow; Lower risk in Mother-Child Transmission	(44, 50)
	B*40^b^	Slow Susceptible	(44) (42)
	B*14 or B*1402	Slow	(24, 46)
	C8	Slow	(24)
	B*81	Slow	(45)
	B*5101	Slow	(47)
	A*02^b^-Cw*16	Increased VL	(51)
	A*02-B*45-Cw*16	Increased VL	
	B*08	Rapid	(8)
	A*24	Rapid	(55)
	A*29	Increased risk in Mother-Child Transmission	(50)
	B*7	Rapid, B-clade infection	(56)
	B*35-Px: B*3502, B*3503, B*3504, B*5301 Putative B*35-Px: B*3509 Putative B*35-Py: B*3505^b^	Rapid Increased VL Increased VL in Peruvians	(54, 58) (59)
		Slow in Thais	(29)
	B*35-Py: B*3501^b^	Slow Increased VL	(61) (62)
	A, B −21M	Rapid	(9)
	DQB1*0603 DQB1*0609, DQB1*050301, DQA1*010201-DQB1*0603, DQB1*0302 DQB1*0602	Resistant Resistant Resistant Rapid; Susceptible	(65, 66) (65) (67) (65, 66)
	DQA1*010201-DQB1*0602,	Susceptible	(65)
	DQA1*0504-DQB1*0201,		
	DQA1*010201-DQB1*0201,		
	DQA1*0402-DQB1*0402,		
	DQA1*0402-DQB1*030101		
	DR2	Susceptible; Susceptible to HIV+PTB	(42) (68)
	HLA-B*35	Increased risk in superinfection	(69)
	HLA-C*04,	Increased risk in superinfection	
	HLA-DRB1*08	Increased risk in superinfection	
Chemokine	CCR5D32^a^	Resistant; Slow	(70–75)
Receptors	CCR5-59029A/G	Slow	(76, 77)
	CCR2V64I	Slow; Less susceptible	(78, 79)

a *Protective effect deduced from multiple independent studies*.

b *Controversial roles in disease progress*.

### Chemokine receptors involved in the impairment of HIV-1 infection

HIV-1 virus invades host CD4^+^ T cells via interactions between CD4 and chemokine receptors on the target cells and the corresponding CD4 and co-receptor binding sites on the viral envelope. Variations in the genes encoding chemokine receptors and their natural ligands influence the rate of HIV-1 disease progression (Table 1). Both innate (monocytes and macrophages) and adaptive immune cells (T cells) express chemokines receptors, and play crucial roles in HIV-1 virus entry and progression.

Chemokines are classified into four subfamilies: C, CC, CXC, and CX3C (80). HIV T-tropic or X4 strains preferentially infect T cells, whereas M-tropic or R5 strains infecting both macrophages and T cells. CCR5 (R5-tropic) and CXCR4 (X4-tropic) are the two main chemokine receptors involved in HIV infection. CCR5 is the major co-receptor for binding of HIV-1 envelope, and thus mediates membrane fusion and signal transduction. CCR5 and CCR2 have been implicated in the HIV-1 transmission and progression of HIV infection. A 32-bp deletion mutation in the CCR5 open reading frame (ORF) (CCR5Δ32) found in Caucasians leads to a frameshift, resulting in abrogated expression of the receptor on the cell surface. Strikingly, homozygosity for CCR5Δ32 confers nearly complete protection against HIV infection (70). A survival analysis revealed that disease progression is slower in CCR5Δ32 heterozygotes than in CCR5–wild-type (WT) homozygotes (71), and this observation was reproduced in other studies. For instance, one study showed that reduced cell surface CCR5 in CCR5 WT/Δ32 heterozygotes yielded limited protection, whereas Δ32 homozygotes were almost completely resistant to HIV infection (72). Meta-analysis of 5,963 HIV-infected individuals and 5,048 controls confirmed that among Caucasians, the risk of HIV infection is at least 13% lower in CCR5 WT/Δ32 heterozygotes than in CCR5 WT/WT homozygotes (73). Additional evidence for the protective role of the CCR5Δ32 allele is provided by the population of Vlach Gypsies in Hungary, in which the frequency of CCR5Δ32 is elevated and the incidence of HIV/AIDS is lower (74). The most famous case of CCR5Δ32 is the “Berlin patient” who was the first and only patient cured of HIV by receiving stem cells homozygous for CCR5Δ32 during treatment of acute myeloid leukemia (AML) (75). The Berlin patient has been free of ART with undetectable HIV since 2008, when he underwent transplantation.

As with HLA, SNPs of CCR5 impact HIV disease progression. Genetic polymorphism CCR5-59029A/G, an A-to-G substitution at position 2459 upstream of the CCR5 translation start site in the gene's promoter region, slowed progression to AIDS by an average of 3.8 years (*p* = 0.004). The protective effect may be the result of reduced *CCR5* mRNA production: *in vitro* promoter activity measurements revealed that 59029G was 45% less active than 59029A (76). However, the association of CCR5-59029A/G with host resistance to HIV-1 infection was not detected in Chinese Han and Indian population (81, 82). The CCR5-59356C/T polymorphism had a mixed effect on disease progression in terms of CD4^+^ counts and VL (77, 82), possibly due to the genetic profiles of the ethnicities involved. *In vitro* functional assays of several SNP of CCR5, including C20S, C178R, A29S, L55Q, C101X, and FS299 (83) resulted in aberrant expression on the cell surface, alteration of ligand binding affinity, or inability to mediate receptor activation, all of which could influence disease progression *in vivo*.

Expression of CCR2, which mediates attraction of monocytes to inflamed tissue (84) is relatively restricted to certain types of cells, mainly circulating monocytes. In addition, CCR2 serves as a minor HIV co-receptor, although the tropism has not been precisely defined. CCR2V64I or 46295G/A, a mutation in the first transmembrane region, is common in all ethnic groups. A genetic association analysis of 1746 AIDS patients revealed a 30–80% increase in the CCR2V64I allele frequency in virus controllers. In a combination of five AIDS cohorts constituting a total of 3003 patients, CCR2V64I was associated with 2–4 years postponement of AIDS relative to homozygotes for the other allele; however, disease delay was not linked to CCR2 genotype in the ALIVE cohort, which consisted of 94% African Americans (78). Nonetheless, the association of CCR2V64I with lower susceptibility to HIV-1 infection has been observed in a cohort of African Americans (79). *In vitro* experiments suggested that the protective effect of CCR2V64I is likely mediated by binding to CCR5 in the cytoplasm, preventing it from being expressed on the cell surface (85). Genetic variation in CXCR2, a receptor for IL-8, has not been significantly associated with HIV-1 VL or CD4 count, although the CXCR2 (+1208) T/C polymorphism is linked to the incidence of opportunistic infections among HIV patients in South Africa (86).

In summary the relationship between chemokine receptors and the mechanisms of disease progression primarily involves the expression of CCR5, which mediates viral binding to the cell surface. Accordingly, reducing CCR5 expression on the cell surface should decrease the risk of viral exposure.

### T cells specific to HIV-1 epitopes

Some healthy individuals at high risk exposure or elite controllers/LTNPs harbor no protective HLA or CCR alleles that could explain their immunity to HIV. Both CD8^+^ and CD4^+^ T cells can be involved in delayed progression to AIDS and suppression of viral replication in the absence of ART (Table 2). In comparison with HIV progressors, LTNPs have HIV-specific CD8^+^ T cells with superior cytotoxicity (101, 102), which suppress HIV replication of autologous HIV-infected CD4^+^ T cells *in vitro* (102, 103). These effects involve upregulation of perforin and loading of lytic granules in CD8^+^ T cells (102, 104, 105). CD8^+^ T cells from elite controllers exhibit more sustained effector properties than those of chronic progressors (106).

**Table 2:** The influence of cell surface proteins of T cells on the outcome of HIV-1 infection.

	**Genes or products**	**Population**	**Phenotypes**	**References**
CD8^+^	Dominant TCR Clonotypes	Elite; LTNP	Better duration; Cross reactivity	(87–89)
	KK10-specific TCR clonotype BV27-CASSGGRRAF/J1-1	Elite vs. Progressors (4% vs. 20%)	Less effective	(87)
	KK10-specific TCR clonotype BV21-CASTNRGSEQY/J2-7	Elite vs. Progressors (4% vs. 17.7%)	Less effective	(87)
	Preservation of CD73 on cells	Elite	Normal function	(90)
	Up regulation of PD-1	LTNPs vs. TP (16.3% vs. 45.2%)	Reduced CD4^+^ T cells; Increased VLs	(91, 92)
	Increase of HLA-G^+^	Controllers	Higher CD4^+^ T cells; Lower VLs	(93)
CD4^+^	Gag293-specific TCR clonotype TRAV24	Controller vs. treated patients (44% vs. 13%)	Superior functions	(94)
	Gag293-specific TCR clonotype TRBV2	Controller vs. treated patients (82% vs. 28%)	Superior functions	(94)
	CD45RA^+^CCR7^−^	LTNPs	IFN-γ secreting	(95)
	Depletion of CD73^+^ on cells	Progressors	Reduced CD4^+^ T cells	(96)
	Frequency of Tregs of CD45RA^+^CD27^−^CCR7^−^ CD62L^−^ and CD45RA^−^CD27^−^CCR7^−^CD62L^−^	ART^−^ population	Higher VLs within total CD4^+^ T cells	(97)
	CTLA-4 on Tregs	Infected population	Corresponding to stages of disease	(98)
	ICOS on Tregs	Infected population	Corresponding to stages of disease	(98)
	Steady CD39 on Tregs	Elite	Stable CD4^+^ T cells;	(98)
	Preserved Vγ2Vδ2 β7^Hi^	NVSs Progressors	Suppressed VLs; Higher CD56 Higher risk of infection; More rapid progression	(99) (100)

CD8^+^ T-cell recognition of virus-infected cells is restricted by host major histocompatibility complex (MHC) class I. Consequently, the protective effects of HIV-specific CD8^+^ T cells in HIV-1 controllers, who have lower VLs and slower disease progression, is due in part to certain MHC class I alleles, such as HLA-B^*^57 and HLA-B^*^27. HIV-1 mutational escape, driven by the immune pressure of CD8^+^ T-cell targeting of the conserved Gag epitope, is detrimental to viral fitness in the presence of protective HLA alleles that decrease viral replicative capacity (107). For instance, the N271H mutation located in the immunodominant HLA-B^*^27-restricted p24 Gag epitope KK10 results in a 40% reduction of infectivity relative to wild-type Gag (4). T-cell receptor (TCR) clonotypes and the avidity of CD8^+^ T cells are also associated with anti-HIV efficacy (87, 108). A longitudinal study of KK10-specific CD8^+^ TCR repertoires in a cohort of HLA-B^*^2705 LTNPs suggested that these individuals could rearrange the TCR to yield high affinity for viral epitopes (88).

Many HIV progressors harboring protective alleles are unable to control HIV infection, leading researcher to attempt to characterize TCR in individuals expressing the same HLA allele but exhibiting different HIV infection phenotype. In five elite controllers and five progressors infected by virus with the KK10 epitope, quantification of KK10 epitope–specific CD8^+^ T cells by staining with HLA-B^*^27–KK10 tetramer revealed no significant difference in the proportion of KK10-specific cells between the two groups (87). However, antigen-specific CD8^+^ T cells from HIV-1 controllers had better functional activity, including greater potency in loading and delivery of perforin to inhibit HIV-1 replication and broader cross-recognition of HIV-1 viral variants (87). Depleting KK10-specific CD8^+^ T cells from bulk CD8^+^ T cells in controllers led to a 90% reduction in the inhibition of HIV replication in autologous CD4^+^ T cells, indicating that immune control was mediated by a KK10-specific response and demonstrating the functional importance of CD8^+^ T cells. Further detailed analysis revealed that the superior ability of CD8^+^ T cells in controllers was correlated with dominant TCR clonotypes. Despite considerable heterogeneity in TCR gene usage among people targeting genetically identical epitopes through genetically identical HLA alleles, dominant clonotypes of KK10-specific CD8^+^ T cells were diversely distributed between elite controllers and chronic progressors, as determined by TCR sequencing of cloned KK10-specific CD8^+^ T cells derived from sorted KK10 tetramer–positive cells. The same two clonotypes, TCRBV27-CASSGGRRAF/J1-1 and TCRBV21-CASTNRGSEQY/J2-7, were identified in two subjects from elite controllers and progressors; nonetheless, the frequencies in individuals were 4% vs. 20% and 4% vs. 17.7%, respectively. The capacity of these clonotypes to recognize HIV-1 and viral variants varied considerably, with the most effective immunodominant clonotypes derived from controllers (87). Superior clonotypes from controllers with the ability to cross-recognize of dominant epitope variants prevented the emergence of viral escape mutants (89).

HIV-1 infection is characterized by host immune system dysfunction represented by chronic immune activation, suppressed T lymphocyte functions, and immune exhaustion. CD39 and CD73 are an ecto-ATPase and an ecto-5′-AMP nucleotidase, respectively; both are broadly expressed by a variety of immune cells. CD39 degrades ATP and ADP to AMP, and CD73 further degrades AMP to adenosine (ADO) (109), and is therefore potently immunomodulatory (110). Down-regulation of CD73 in T cells is correlated with immune activation and functional defects in the T cells of HIV-infected people (90, 111, 112). Preservation of HIV-specific CD73^+^ CD8^+^ T cells is a characteristic feature that distinguishes HIV elite controllers from HIV-1 progressors (90).

Antigen (Ag)-specific CD8^+^ T cells are among the major components involved in controlling HIV infection, but eventually these functional CD8^+^ T cells lose their antiviral activity. Inhibitory programmed death-1 (PD-1) receptor, an exhaustion marker, is also relevant to HIV disease progression (91, 92). Up-regulation of PD-1 is partially responsible for loss of control of virus replication due to CD8^+^ T-cell dysfunction, e.g., reduced production of perforin and IFN-γ. PD-1 up-regulation on total and HIV-specific CD8^+^ T cells correlates significantly with reduced CD4^+^ T cell numbers and elevated plasma HIV-1 load in both LTNPs and TP patients. However, LTNPs have not only significantly fewer PD-1 HIV-specific CD8^+^ T cells than TP patients (16.3%) vs. 45.2%, *P* <.05) (92), but also express significantly lower levels of PD-1 on HIV-specific CD8^+^ T cells (91, 92). Median fluorescence intensity (MFI) analysis revealed that PD-1 expression on HIV-specific CD8^+^ T cells varied among different populations of Ag-specific CD8^+^ T cells in comparison with donor-matched naive cells. The heterogeneity in PD-1 expression among different virus-specific CD8^+^ T cell populations in elite controllers implies that the Ag-specific CD8^+^ T cells have lost their ability to fully suppress PD-1 transcription (91). The subset of CD4 and CD8 T cells expressing the non-classical HLA class Ib molecule HLA-G has regulatory properties (113). The proportion of HLA-G^+^ HIV-1-specific CD8 T cells is higher in HIV-1 controllers than in disease progressors; the frequency of these CD8^+^ T cells is directly associated with CD4 T-cell counts and inversely correlated with VLs, indicating an association with HIV-1 immune control (93).

In the ANRS CODEX cohort, HIV controller (HIC) Gag-specific CD4^+^ T cells consistently exhibited superior IFN-γ production and degranulation capacity in comparison with long-term–treated patients (114). Gag293 peptide is the most immunoprevalent CD4 epitope. Using a device allowing the amplification of MHC II tetramer–positive cells in short-term primary CD4^+^ T-cell cultures, a population of CD4^+^ primary T cells with high antigen sensitivity and high MHC II tetramer binding capacity for Gag293 peptide was identified in HICs; however, such cells were absent in ART-treated patients (114). Titration of the Gag293 peptide for intracellular cytokine staining (ICS) response confirmed the properties of Gag-specific CD4^+^ T cells in HICs, which were distinguished from those in ART-treated patients by their superior proliferative function and IFN-γ-secreting effectors. TCR features may explain the remarkable performance of HIV-specific CD4 responses in controllers, because neither HLA genetic background nor residual virus could explain the superior activity. The high-affinity Gag293-specific TCRs were cross-restricted by as many as five distinct HLA-DR alleles of diverse genetic backgrounds in HIV controllers, and both HIV controllers and ART recipients had long-term undetectable VL (>10 years) and were therefore unable to drive the induction of high-affinity TCR. Analysis of the TCRα variable gene (TRAV) and TCRβ variable gene (TRBV) of these CD4^+^ primary T cells revealed biased preferential gene usage. TRAV expression in Gag293-specific CD4^+^ T cells from controllers was highly skewed, with a median of 44% of cells expressing the TRAV24, whereas this gene family was amplified at much lower levels in cells from treated patients (13%; *P* = 0.037). TRBV2 was amplified in Gag293-specific cells from the vast majority of controllers (82%, vs. 28% in treated patients). Public clonotypes of TRAV24 and TRBV2, defined as identical CDR3 AA sequences found in at least two individuals without any mismatch, were significantly more frequent in the HIC group than in the ART group. Transfer of these public clonotypes into healthy donor CD4^+^ T cells and coculture with autologous monocyte-derived dendritic cells (MDDCs) pulsed with Gag293 conferred high antigen sensitivity and polyfunctionality, including multiple cytokine production and degranulation capacity. Thus, transduction of public clonotypes of TRAV24 and TRBV2 recapitulated key features of the controller CD4 response (94).

Harari et al. (95) reported phenotypic heterogeneity (CD45RA^−^CCR7^+^, CD45RA^−^CCR7^−^ and CD45RA^+^CCR7^−^) of HIV-specific CD4 T cells among LTNPs, representing protracted Ag exposure and low VL. Chronic stimulation with low Ag levels resulted in the appearance of memory CD4 cells with the CD45RA^+^CCR7^−^ phenotype that secreted IFN-γ when being stimulated with HIV-1 Gag p55; by contrast, the same population in HIV progressors had no IFN-γ-secreting activity. The CD45RA^+^CCR7^−^ cell population is at an advanced stage of differentiation, as evidenced by upregulation of the surface marker CD57 (95).

The typical stages of HIV infection are gradual depletion of CD4^+^ T cells, immune activation, and exhaustion. The immune regulatory molecule CD73 may play a crucial role in controlling HIV-1–associated immune activation. Among 36 HIV-1–positive individuals treated and not treated with ART, depletion of the ADO-producing CD4^+^CD73^+^ subset of T cells accounted for the loss of suppression of responder cell proliferation (96). Absolute number and frequency of CD4^+^CD73^+^ T cells positively correlated with CD4^+^ T cell count, regardless of viral suppression, and negatively correlated with expression of CD38 (representing activated CD4 T cells) and inflammatory C reactive protein (CRP) expression in plasma (96). T-cells of the CD4^+^CD73^+^ subset belong to the memory compartment (CD45RO^+^) and are not classified as regulatory T cells (Tregs) because they do not express the commonly used Treg markers CD25 and FOXP3 (96).

Tregs expressing the classic markers CD25 and FOXP3 are also involved in the coordination of immune responses and maintenance of homeostasis. Assessment of the frequency and quality of regulatory T cells (CD4^+^CD25^+^CD127^low^) from a cohort of 31 HIV-infected ART-naïve patients displayed dominant phenotypes by effector (CD45RA^+^CD27^−^CCR7^−^ CD62L^−^) and effector memory (CD45RA^−^CD27^−^CCR7^−^CD62L^−^) cells with enhanced expression of CD39, CD73, HLA-DR, and CD38, differing from HIV-negative individuals who mainly displayed naive (CD45RA^+^ CD27^+^ CCR7^+^ CD62L^+^) and central memory (CD45RA^−^CD27^+^ CCR7^+^ CD62L^+^) cells. The total number of Treg cells inversely correlated with HIV VL and positively with CD4^+^ count. However, among total CD4^+^ T cells, Treg cell frequency correlated positively with HIV-1 plasma VL (97), consistent with the frequency and phenotype of Tregs in a well-characterized larger cohort of 131 HIV-infected patients. Patients with high HIV-1 viremia and progressive disease at different stages exhibited a significant elevation in relative Treg frequency within the CD4^+^ compartment, whereas patients with elite status had Treg frequencies relatively comparable to those of healthy individuals (98). Two surface molecules, the cytotoxic T-lymphocyte-associated antigen 4 (CTLA-4) and inducible costimulator (ICOS), which function in controlling adaptive immune responses, were expressed at significantly higher levels in Treg cells than in healthy controls, although the levels of expression differed according to disease stage. A significant increase in CD39 expression was observed in Treg cells of patients, but not in the healthy or elite populations (98).

γδ T cells serve as a bridge between innate and adaptive responses (115). Quantitative changes in Vγ2Vδ2 T cells after HIV infection were unique among cohorts, including natural viral suppressors who have undetectable VLs for >2 years without ART (NVSs, *n* = 21), HIV negative individuals (HIV-, *n* = 27) and HIV viral controllers on ART (HIV-P, *n* = 25). The proportion of Vδ2^+^ was significantly higher among NVSs than in the HIV- and HIV-P groups. The frequency of Vγ2 chains was lower among NVSs than in HIV-controls (*P* = 0.016) but higher than in HIV-P patients (*P* = 0.03), indicating that NVSs better preserved Vγ2Vδ2 T cells. In addition, Vγ2^+^ cells of NVSs exhibited sustained function by expressing higher levels of CD56, and comparable or higher CD56 expression post isopentenyl pyrophosphate (IPP) stimulation compared to HIV- and HIV-P groups (99).

Integrin α4β7 promotes homing of T cells to gastrointestinal (GI) mucosa. α4β7 directs CD4 T cells to the α4β7 binding site in the V2 region of some viruses, which facilitates their attachment to target CD4 T cells. α4β7 integrin expression is associated with HIV clinical outcomes (100). Remarkedly, frequencies of β7^Hi^ CD4^+^ T cells predicted 17% (*P* = 0.007) increased risk of HIV acquisition and more than twice rate of CD4 T cell counts progression to < 500/μl in a cohort of high-risk African women.

Overall, the ultimate functionality of T cells signifies their effect on HIV infection outcome. In disease controllers, CD4^+^ and CD8^+^ specific T cells contain either higher frequency of the most effective clonotypes or lower frequency of the less effective clonotypes, e.g., Gag293-specific immunodominant TCR clonotype TRAV24 and TRBV2 on CD4^+^ T cells and the KK10-specific TCR clonotype BV27/J1-1 and BV21/J2-7 on CD8^+^ T cells, respectively. Superior function of T cells implies preservation of certain phenotypic or constitutive T cells modulated by immune regulatory molecules (mainly CD73 and CD39) on the cell surface. In addition, certain CD4^+^ T cell phenotypes that promote HIV attachment and invasion, such as β7^Hi^, predict the risk of infection and disease progression.

### B cell–relevant IgG allotypes and allelic Fc receptors linked to disease progression

HIV-1 infection not only results in hyperactivation, dysfunction, and immune exhaustion of T cells, but also stimulates B-cell hyperactivation and dysfunction, reflected by hypergammaglobulinemia and lack of pathogen-specific Ab responses (116–118). In contrast to HLA alleles, the influences of genetically and phenotypically distinct B-cell populations on the progression of HIV-1 infection progression remains virtually unexplored. gp41 IgG2 Abs are present at higher levels in elite controllers than in non-controllers (*p* = 0.03) (119), and enrichment of p24 IgG1 Abs in chronic HIV-1 subtype C infection independently predicts low VL (*P* = 0.04) and high CD4 cell counts (*P* = 0.004). Because these evaluations were adjusted for the impact of Gag-specific T-cell responses and protective HLA class I alleles, this viral control is likely to be mediated by higher levels of antibody-dependent cellular phagocytosis (ADCP) and antibody-dependent cellular cytotoxicity (ADCC), (120, 121). Overall, broadly neutralizing antibody (NAbs) responses are not particularly associated with LTNPs except in the case of glycan-dependent NAbs, which are significantly more abundant in LTNPs than in TPs (*p* = 0.0017) (122). Over the course of infection, increasing levels of Env-specific IgG2 Abs concomitant with decreasing levels of Env-specific IgG3 Abs are associated with loss of HIV infection control (123). The associations between HIV disease progression and B-cell membrane markers such as CD39 and CD73 have been examined (124), and a few other studies investigated the relationship between HIV progression and genetically different allotypes of immunoglobulin heavy chain (GM), kappa light chain (KM) (genetic marker), and Fc receptors (FcRs) (125, 126). FcRs are Ab-related and surface proteins present on many leukocytes, including B lymphocytes, follicular dendritic cells, natural killer cells (NK cells), macrophages, and neutrophils (Table 3).

**Table 3:** B cells and B cells related Ig and FcR genotypes relevant to the outcome of HIV-1 infection.

**Genes or products**	**Population**	**Phenotypes**	**References**
Enriched gp41 IgG2^b^ Enriched p24 IgG1^a^	Elite Chronic subtype C infection	Higher ADCP and ADCC	(119) (120, 121)
Glycan-dependent NAbs	LTNPs		(122)
Increased Env-specific IgG2^b^ plus Decreased Env-specific IgG3	Losing infection control		(123)
Higher IgG2 to gag; Higher IgG1 to p32	Controllers carrying no protective HLA-B alleles		(127)
Increased IgG1 in FcgRIIa-binding immune complexes	Non-controllers		(127)
IgG to p24^a^	HIV controllers carrying no HLA-B*5701	Stronger opsonophagocytosis	(128, 129)
Decreased CD39/CD73 on CD20 cells	Viremic progressors	Increased proliferation and exhaustion; Ab class switch	(124)
GM21 non-carriers within FcγRIIa R^b^ non-carriers at position 131; GM21 non-carriers within FcγRIIIa V^b^ allele carriers at position 158	Controllers >Non-controllers	Epistasis	(126)
KM1/3-GM3/17 interaction	Caucasians (vaccine trial)	Epistasis; Risk in HIV acquisition	(125)
KM1/3-GM5/21 interaction	All participants (vaccine trial)	Epistasis; Risk in HIV acquisition	(125)
GM23+/–FcγRIIIa interaction	Caucasians; All participants (vaccine trial)	Epistasis; Risk in HIV acquisition	(125)
FcγRIIa RR^b^ at position 131	Progressors (MACS cohort) (CD4 cell count <200/mm3)	Less affinity to IgG; Less internalization	(130)
FcγRIIIa VV^b^; FcγRIIa RR: FcγRIIIa FF	Progressors Progressors	Higher affinity to Fc; Immune activation	(131) (131)

a *Protective effect deduced from multiple independent studies*.

b *Controversy in distribution in infected populations*.

Assessment of plasma IgG1 and IgG2 Abs from 32 HIV controllers and 21 non-controllers with and without protective HLA-B alleles (HLA-B^*^57, HLA-B^*^27, HLA-B^*^1402+Cw0802 or HLA-B^*^52) revealed higher plasma levels of IgG1 Abs against HIV Gag (p18, p24, rp55), Pol-encoded (p32, p51, p66) proteins, and gp120 in HIV controllers. Interesting, HIV controllers not carrying a protective HLA-B allele had significantly higher levels of IgG2 against Gag protein and IgG1 against p32, whereas IgG1 levels were significantly higher in FcgRIIa-binding immune complexes from HIV non-controllers than in HIV controllers (127). Moreover, in the same group, the association between HIV-1 p24-specific IgG Abs and natural control of HIV-1 infection in individuals not carrying HLA-B^*^5701 was only observed in HIV controllers, but not in elite controllers or non-controllers (>10,000 copies/mL) (128). HIV-1 p24-specific IgG Abs exhibited higher plasmacytoid dendritic cell (pDC)-reactive opsonophagocytosis in HIV controllers and elite controllers than in non-controllers (129).

The immune modulators CD39 and CD73 are widely expressed on a wide range of immune cells, including B cells. They are recognized as B-cell differentiation markers (132) and play critic role in B cell–mediated suppression of T-cell functions (133). CD73 is also important for initiation of Ig class switching (134) and adhesion of B cells to follicular dendritic cells in the germinal center (135); consequently, it serves as a migration marker. Kim et al. (124) reported significant down-regulation of CD39/CD73 on CD20^+^ B cells in 70 patients with HIV (all stages of disease) relative to 21 healthy individuals. Down-regulation of CD39/CD73 might be a characteristic of HIV infection, as expression of these proteins was not altered in the same population of B cells from 16 patients infected with hepatitis C virus (HCV). Reduced expression of CD39/CD73 was not observed at different stages of elite, LTNP, and ART patients, suggestive of correlation with disease progression (124). The authors of that study further analyzed CD39/CD73 expression across all B-cell subsets—naïve B cells (CD21^+^, CD27^−^), resting memory B cell (CD21^+^,CD27^+^), active memory B cell (CD21^−^,CD27^+^), and tissue-like memory B cells (CD21^−^,CD27^−^) in dissected groups—and observed that expression of CD39/CD73 was markedly lower than in healthy individuals or ART patients, as in the case of CD20^+^ B cells overall. A significant difference in CD73 expression was also observed in viremic vs. aviremic patients with HIV. However, there was correlation between CD4^+^ counts and the number of CD39^+^CD73^+^ co-expressing cells, while VL was not correlated with these cells (124). Reductions in the levels of CD39/CD73 on the B-cell surface were functionally correlated with increased B-cell activation and *in vitro* impairment of AMP consumption, as well as, Ab class switching (124).

Allotypes expressed on the constant region (Fc) of IgG heavy chain and kappa chain are designated as allotypes GM and KM. GM comprises the G1M, G2M, and G3M allotypes representing the human IgG1, IgG2, and IgG3 molecules. To date, 18 GM allotypes have been identified: four G1M, one G2M, and thirteen G3M. Similarly, KM allotypes are designated as KM1, KM2, and KM3. The allotypes are inherited in fixed combinations within different populations (136). Antibody segment Fc mediates ADCP, ADCC, antibody-dependent cell-mediated virus inhibition (ADCVI), and antibody-dependent complement–dependent cytotoxicity (ADCDC) via binding to the corresponding Fc receptors (Fcγs) on the surface of effector monocytes, macrophages, dendritic cells, or natural killer cells. Pandey et al. (125) have identified particular GM and Fcγ alleles that contribute, mainly epistatically, to HIV-1 acquisition and suppression. In comparison with GM21 carriers within the FcγRIIa arginine (R) noncarriers at position 131 or within the FcγRIIIa valine (V) allele carriers at position 158, GM21 noncarriers had great odds being in virus controllers (*p* = 0.0214; *p* = 0.0495, respectively). Assessment of epistasis between FcγRIIa and GM21 in a logistic regression model revealed a statistically significant interaction. Genotyping of the FcγRIIa, FcγRIIIa, GM, and KM alleles from 777 participants in a randomized trial of recombinant adenovirus HIV-1 vaccine revealed epistatic involvement of several allotypes in the acquisition of HIV infection: KM1/3-GM3/17 in Caucasians (*p* = 0.0246), KM1/3-GM5/21 (*p* = 0.0016) in all participants, and GM23+/–FcγRIIIa (*p* = 0.0060; *p* = 0.0085) in all participants (125, 126). Accordingly, the authors proposed that the mechanisms underlying spontaneous immune control of HIV-1 might depend on the epistatic interaction between GM and HLA alleles and involve recognition of HIV antigens by allotypically disparate IgG receptors on B cells, followed by processing and presentation on the peptide-binding grooves of protective HLA alleles. To support this idea, they referenced suggestive evidence from (119) regarding the influence of HLA alleles on the magnitude of anti-gp41 IgG2 antibody responses (137).

Polymorphisms in FcγR genes or alleles are associated with progression of HIV infection. The various IgG subclasses have different binding affinities to individual Fc receptors. An SNP of FcγRIIa (CD32) resulting in histidine (H) at amino acid position 131 yields a protein with higher IgG binding affinity than the variant with R at this position (138). In order to investigate the association of FcγRIIa alleles with HIV disease progression, a total of 559 HIV-infected males in the Multicenter AIDS Cohort Study (MACS) were genotyped for FcγRIIa. Compared to subjects with any H allele (i.e., HH or HR), homozygous RR subjects were significantly more likely to have CD4^+^ cell counts <200/mm^3^ (130). However, the association of FcγRIIa RR alleles with HIV disease progression was not confirmed in studies of cohorts dominated by African Americans (131) or consisting entirely of African Kenyan women (131, 139). Measurements of the frequency of monocytes containing immune complex (IC) of FITC-labeled HIV-1 virion and polyclonal HIV-positive IgG revealed that monocytes from HH homozygous subjects exhibited higher rates of internalization of antibody–virion complexes. This active internalization was mediated by IgG2, as demonstrated by the observation that depletion of IgG2 from polyclonal HIV-positive IgG decreased internalization by monocytes from HH donors, but had little or no impact on cells from RR donors (130).

Another common allelic variant V in FcγRIIIa conferred higher affinity to Fc than allelic phenylalanine (F) (138). In a small cohort study of 59 natural HIV progressors and 43 natural virus suppressors (NVSs), the VV genotype was not found among NVSs, and was rare among uninfected controls; instead, the vast majority of VV carriers (95%) were HIV progressors. The higher affinity of FcγRIIIa VV genotype induced immune activation in progressors, which was incongruent with the protective effect in GM21 noncarriers-FcγRIIIa VV carriers (126), yet it was unclear about the corresponding GM allele in the progressors. FcγRIIa alleles were not relevant to disease progression in that small cohort, although RR:FF double homozygous genotype was linked to HIV progression. Of 59 HIV progressors, 11 had this combined genotype, vs. 1 of 40 NVSs and 3 of 34 HIV-negative controls (131).

A handful of B-cell surface proteins, Ig, and FcR have been associated with disease progression to date. As with CD39/CD73 on T cells, surface proteins on B cells are linked with disease control via immune regulation, and the allotypes of Ig and FcγR alleles on effector cells act epistatically on the ultimate outcome. Elevated levels IgG2 Abs are observed in both controllers and non-controllers. Accordingly, it may be necessary to carefully analyze the influence of G2M alleles and their cognate counterparts on effectors.

### Suppressive effect of NK cells on HIV infection

Not all HIV-1 controllers carry protective HLA-B alleles or readily exhibit HIV-specific CD8^+^ T-cell responses (140). The controller status of these individuals can be explained by alternative mechanisms of immune control mediated by natural killer (NK) effector responses (Table 4).

**Table 4:** The influence of cell surface proteins of NK cells on the outcome of HIV-1 infection.

**Genes or products**	**Population**	**Phenotypes**	**References**
KIR 3DL1*h/*h or *004 + HLA-B*57	More frequent in exposed uninfected individuals		(141)
Increased KIR2DL2/L3/S2; Decreased KIR2DL1/S1	Infected patients	Increased VLs	(142)
KIR3DL1^a^	Slow progressor	Lower VLs; Strong NK responses	(22, 143, 144)
KIR3DS1^b^+HLA-B Bw4-80I (irrespective of HLA-B*57or HLA-B*27)	Slow progressors	Epistasis NK cell function	(145) (146)
KIR3DS1^b^	Progressors	Rapid to endpoint	(145)
HLA-C*01:02+KIR2DL2; HLA-C*12:02^b^+KIR2DL3; HLA-B*46:01+KIR2DL2	CRF01_AE chronically infected Thais	Higher VLs	(147)
HLA-C*12:03+KIR2DL2; HLA-C*12:03+KIR2DS2	CRF01_AE chronically infected Thais	Lower VLs	(147)
Increased NKp44 (CD56dim)	Clade A/D infected Ugandans	Reduced CD4^+^ T cells	(148)
No induction of NKp44 Upon rIL2 stimulation	EC/LTNPs	CD4 maintenance	(149)
Lower NKp30 upon rIL2 stimulation	ECs and LTNP		(149)
NKG2A	Infected population	Higher VLs; Reduced CD4^+^ T cells; Increased NK inhibition	(9)

a *Protective effect deduced from multiple independent studies*.

b *Controversial roles in disease progress*.

NK cells play important roles in both the innate and adaptive immune responses. Concerning the former, NK cells recognize and quickly respond to viral-infected cells or tumor cells in the absence of Abs and MHC. NK cells inhibit viral replication *in vitro* by secreting chemokines that compete for binding to R5 viruses (150) or by promoting lysis of HIV-1–infected autologous CD4^+^ T cells (151, 152). A 3–5 years follow-up study of the relationship between NK cell function and outcome among Vietnamese intravascular drug users (IDUs) at high risk for HIV exposure indicated that NK cells contribute to protection against HIV (153). NK cells from exposed uninfected (EU) IDUs had significantly higher levels of cytokine secretion and lytic activity than unexposed controls or IDU seroconverters, although the percentage of NK cells among PBMCs did not differ among the three groups (153), supporting a role for genetic background of NK cells. Genetic protection by NK cells was also evidenced by the reduced risk of HIV infection in individuals carrying a combined genotype of HLA-B^*^57 and the KIR 3DL1^*^h/^*^h or KIR3DL1^*^h/^*^004 alleles, which express high levels of KIR3DL1. This combined genotype was more frequent in EU individuals (12.2%) than in individuals with primary infection (2.7%) (*P* = 0.019) (141).

Activating and inhibitory receptors are present on the membranes of NK cells. In general, natural cytotoxicity receptors (NCRs), NKG2(CD94), and FcγIIIa (CD16) are activating receptors, whereas inhibitory receptors include killer-cell immunoglobulin-like receptors (KIRs) and leukocyte inhibitory receptors (LIR). KIR receptors of NK cells are differentiated by the number of extracellular immunoglobulin domains and the length of the cytoplasmic tail: KIR2D and KIR3D have two and three extracellular domains, respectively; proteins with long intracellular tails 1–3 (L1–3) mediate inhibitory signals, whereas those with short intracellular tails (S) mediate activating signals with or without associated adaptor proteins. KIR genes appear to have variegated expression, and their corresponding cognate HLA class I ligands are highly variable. Different ethnicities have skewed distributions of KIR and HLA variants (154). KIR molecules interact specifically with HLA-A, HLA-B, or HLA-C. Bw4, a putative ligand for KIR, can be classified into the Bw4-80I (less HLA-B alleles and HLA-A alleles) and Bw4-80T (more HLA-B alleles) allotypes based on the present of isoleucine or threonine at position 80. HLA-C1 and HLA-C2 are ligands to KIR that interact with KIR2DL2/L3/S2 and KIR2DL1/S1 respectively. The associations between KIR genes and delayed AIDS progression have been investigated (22, 155). NK-cell cytotoxicity is higher in LTNPs than in patients with viremic HIV and similar to that in controls, despite a reduction in the overall proportion of NK cells in these individuals (156).

The phenotypic and genotypic profiles of NK cells are associated with HIV infection progression. Increased frequency of NK cells expressing KIR2DL2/L3/S2 and decreased frequency of NK cells expressing KIR2DL1/S1 are correlated with VL in all patients (142). The alleles of KIR3DL1 encode high-expression allotypes (KIR3DL1^*^h), low-expression allotypes (KIR3DL1^*^l), and those with no cell surface expression (^*^004). KIR3DL1^*^h has a higher affinity for Bw4-80I than Bw4-80T, resulting in greater inhibition of HIV (143) and thereby slowing the progression to AIDS via elevated NK-cell polyfunction (22). Stratification of 915 HIV-1–infected subjects from four cohorts revealed that at least part of the protection against HIV in terms of disease progression and VL could be attributed to KIR3DL1. From most protective to least protective, the alleles could be ranked KIR3DL1^*^h/^*^h or KIR3DL1^*^h/^*^004 + Bw4-80I > KIR3DL1^*^l/^*^l, KIR3DL1^*^l/^*^h or KIR3DL1^*^l/^*^004+ Bw4-80I > Bw6/Bw6 (trend *P* = 0.001) (143). The protective KIR3DL1^*^h/^*^y receptor genotype being associated with strong NK responses was inversely associated with HIV-1 Gag-specific CD8^+^ T cell responses among elite controllers (144). Because allele ^*^004 is not expressed on the cell surface, slower progression to AIDS among individuals carrying KIR3DL1^*^004 + Bw4 implies a novel intracellular function for KIR3DL1^*^ 004 or linkage disequilibrium with a neighboring locus that confers protection. The inhibition of KIR3DL1^*^h in the presence of Bw4 in association with slow progression to AIDS and lower VL via augmentation of NK functions seems contradictory. However, this phenomenon may demonstrate a mechanism called NK cell licensing: previous engagement of inhibitory receptor of NK cells with MHC-I molecules would allow for greater responsiveness upon subsequent activation stimuli (157). Bw4 contains the most protective HLA class I alleles, such as HLA-B^*^57 and HLA B^*^27, and the heightened effect of HLA-B^*^57 in combination with distinct KIR3DL1 groups supports the contribution of the allotypes of KIR3DL1 (143). A study of the same cohort of European- and African-Americans revealed that the activating allele KIR3DS1 is also associated with delayed progression to AIDS in conjunction with HLA-B Bw4-80I alleles, independent of the expression of the respective HLA-B Bw4-80I alleles HLA-B^*^57 or HLA-B^*^27 (145). The protective effect was derived from an epistatic interaction between the two loci, as KIR3DS1 alone was associated with rapid progression to AIDS, and HLA-B Bw4-80I only had a protective effect in European-Americans, but no influence on African Americans, who have a lower frequency of KIR3DS1 (*f* = 0.05 vs. 0.22 in European-Americans) (145).

A KIR3DS1 protective role was observed in early HIV-1 infection when they mediated greater effector function inducing IFN-γ and CD107a. The augmented effector activities could not be attributed to Bw4-80I because the levels of IFN-γ and CD107a expression in NK cells did not differ among KIR3DS1 subjects with or without the Bw4-80I allele. NK cells from individuals with at least one copy of KIR3DS1 had higher IFN-γ expression and equivalent CD107a expression in the presence of 721.221 stimulator cells lacking all MHC class I proteins (146). The protective effect of KIR3DS1, associated with slow progression to AIDS, might involve NK cell-mediated inhibition of HIV-1 replication (158).

The effect of HLA-C-KIR2D pairs on disease outcome was recently described in a Thai cohort of ART-naive adults chronically infected with HIV CRF01_AE (147). Subjects with genotypes KIR2DL2/HLA-C^*^01:02, KIR2DL3/HLA-C^*^12:02, and KIR2DL2-HLA-B^*^46:01 had significantly higher VLs than subjects lacking the corresponding KIR2D. Another HLA-C1 allele, C^*^12:03, had supressive effect on VLs when paired with both KIR2DL2 and KIR2DS2.

Significantly increased expression of NKp44, an NCRs on NK cells, was detected in CD56dim NK cells in HIV-1 Clade A– or Clade D–infected Ugandans, and the phenotypic change correlated inversely with absolute CD4 counts, indirectly supporting a role for this receptor in disease progression (148). Upon stimulation by rIL2, expression of NKp44 on NK cells in EC/LTNP was not induced (149), sharply decreasing the ability of NK cells to kill target cells *in vitro*, in contrast to the situation in antiretroviral-treated aviremic progressor patients (TAPPs). The marked impairment of NKp44 inducibility distinguishes HIV controllers (LTNPs/ECs) from progressors, as the lack of NKp44 induction may be related to CD4 maintenance (149). When activated by rIL2, NKp30 was modulated to lower levels in ECs and LTNPs vs. healthy donors (HDs), whereas no induction of NKp30 expression was observed in TAPPs (149). Induction of NKG2D receptor positively influenced ADCC responses, as evidenced by the observation that blockade of NKG2D receptor significantly decreased ADCC activity against infected cells; according to the authors of that study, however, the NKG2D phenotype might be influenced by viral rather than host factors (159). The inhibitory cell receptor NKG2A on NK cells was correlated with VL, CD4 count, and outcome post-infection, as described above in the in HLA section (9). The deleterious effect of NKG2A was mediated through expression of HLA-E, the natural ligand of NKG2A. Enhancement of HLA-E expression due to binding of a signal peptide derived from the leader sequence of HLA-A, -B, and -C molecules promotes NKG2A-mediated NK-cell (and/or T-cell) inhibition. The negative effect of HLA-A on HIV control was exacerbated by HLA-B −21M, which favors NKG2A-mediated NK cell licensing.

In summary, NK cells may impact disease progression independently of, or epistatically with, HLA molecules. Both inhibition and activation of NK cell receptors play roles in control of HIV infection.

## Discussion

The outcome of HIV-1 infection varies dramatically among infected people. The mechanisms underlying slow progression to AIDS in LTNP, especially in elite controllers, has attached a great deal of research interest and inspired numerous studies. A tremendous effort has already been made to solve this puzzle, and the results may eventually bring about a means to conquer AIDS.

Extended studies have revealed that multiple factors are involved in determining HIV-1 disease outcome. In addition to HLA variations, which account for up to 25% of elite controllers, many other elements involving various types of immune cells can contribute to effective immunity to HIV-1. The patterns in which controllers restrain HIV infection vary across populations. Some HLA alleles, for example HLA-B^*^57, slow disease progression by improving presentation of HIV-1 antigens. Other variants, such as CCR5Δ32, allow hosts to resist HIV-1 infection by eliminating components necessary for virus invasion. In addition, some controllers have immune cells, such as CD8^+^ T cells, with superior ability to protect against viruses. In addition, infected individuals may be categorized differently due to intrinsic features of Igs and FcRs on effector cells.

Some protective or deleterious genetic factors are restricted to certain ethnicities and populations, illustrating the importance of genetic identity. For example, CCR5Δ32 is most prevalent in Caucasians. However, genetically inherited immune traits are not the exclusive influence on outcome; in addition, phenotypic traits of immune cells also have an impact on infection status. The preservation of immunoregulatory molecule CD73 on immune cells is a hallmark of slow progressors. Notably in this regard, it is not uncommon for a gene to play contradictory roles in HIV infection, such genes distribute almost each compartment of immune system (Tables 1, 3, 4), which proposes comprehensive association studies.

In light of the modern trend toward personalized medicine, it is especially important to thoroughly investigate how HIV-1 disease is controlled *in vivo*. To date, the complexity of the host immune system is the major challenge in profiling the multifaceted and multifactorial contributions to HIV-1 disease outcome. Successful elucidation of these associations could guide optimal HIV-1 disease treatment and vaccine design within specified genetic populations. The greater efficacy of the RV144 vaccine in the HLA-A^*^02 population highlights the feasibility of personalized vaccine design.

Assessment of cross-sections within individual immune cells is difficult and relies on complete and precise bio-information at different stages of disease. The corpus of previous studies using conventional genotyping methods, particularly those spanning more than 10 years, is relatively limited. With the advent of new technologies, such as genome-wide association studies (GWAS) and whole genome sequencing (WGS), the landscapes of entire gene networks can be elucidated. Recently developed single-cell genome sequencing will allow researchers to establish a high-resolution view of the genes involved, and advances in bioinformatics will provide a powerful tool for efficiently processing the huge amount of information produced by these experiments. It is possible that the puzzles described in this review will be solved in the future following the evolution of sophisticated technologies.

## Author contributions

LL and YL wrote the manuscript, and LL and MG revised the manuscript. All authors have made intellectual contributions to the work and approved it for publication.

### Conflict of interest statement

The authors declare that the research was conducted in the absence of any commercial or financial relationships that could be construed as a potential conflict of interest.
